# Patellar dislocation: cylinder cast, splint or brace? An evidence-based review of the literature

**DOI:** 10.1186/1865-1380-5-45

**Published:** 2012-12-31

**Authors:** Johanna P van Gemert, Lisette M de Vree, Roger A P A Hessels, Menno I Gaakeer

**Affiliations:** 1 Department of Emergency Medicine, University Medical Center Utrecht, Utrecht, The Netherlands

## Abstract

Patellar dislocations are a common injury in the emergency department. The conservative management consists of immobilisation with a cylinder cast, posterior splint or removable knee brace. No consensus seems to exist on the most appropriate means of conservative treatment or the duration of immobilisation. Therefore the aims of this review were first to examine whether immobilisation with a cylinder cast causes less redislocation and joint movement restriction than a knee brace or posterior splint and second to compare the redislocation rates after conservative treatment with surgical treatment. A systematic search of Pubmed, Embase and the Cochrane Library was performed. We identified 470 articles. After applying the exclusion and inclusion criteria, only one relevant study comparing conservative treatment with a cylinder cast, brace and posterior splint remained (Mäenpää et al.). In this study, the redislocation frequency per follow-up year was significant higher in the brace group (0.29; *p* < 0.05) than in the cylinder cast group (0.12) and the posterior splint group (0.08). The proportion of loss of flexion and extension was the highest in the cylinder cast group and the lowest in the posterior splint group (not significant). The evidence level remained low because of the small study population, difference in duration of immobilisation between groups and use of old braces. Also, 12 studies comparing surgical with conservative treatment were assessed. Only one study reported significantly different redislocation rates after surgical treatment. In conclusion, a posterior splint might be the best therapeutic option because of the low redislocation rates and knee joint restrictions. However, this recommendation is based on only one study with significant limitations. Further investigation with modern braces and standardisation of immobilisation time is needed to find the most appropriate conservative treatment for patellar luxation. Furthermore, there is insufficient evidence to confirm the added value of surgical management.

## Introduction

**Figure 1:**
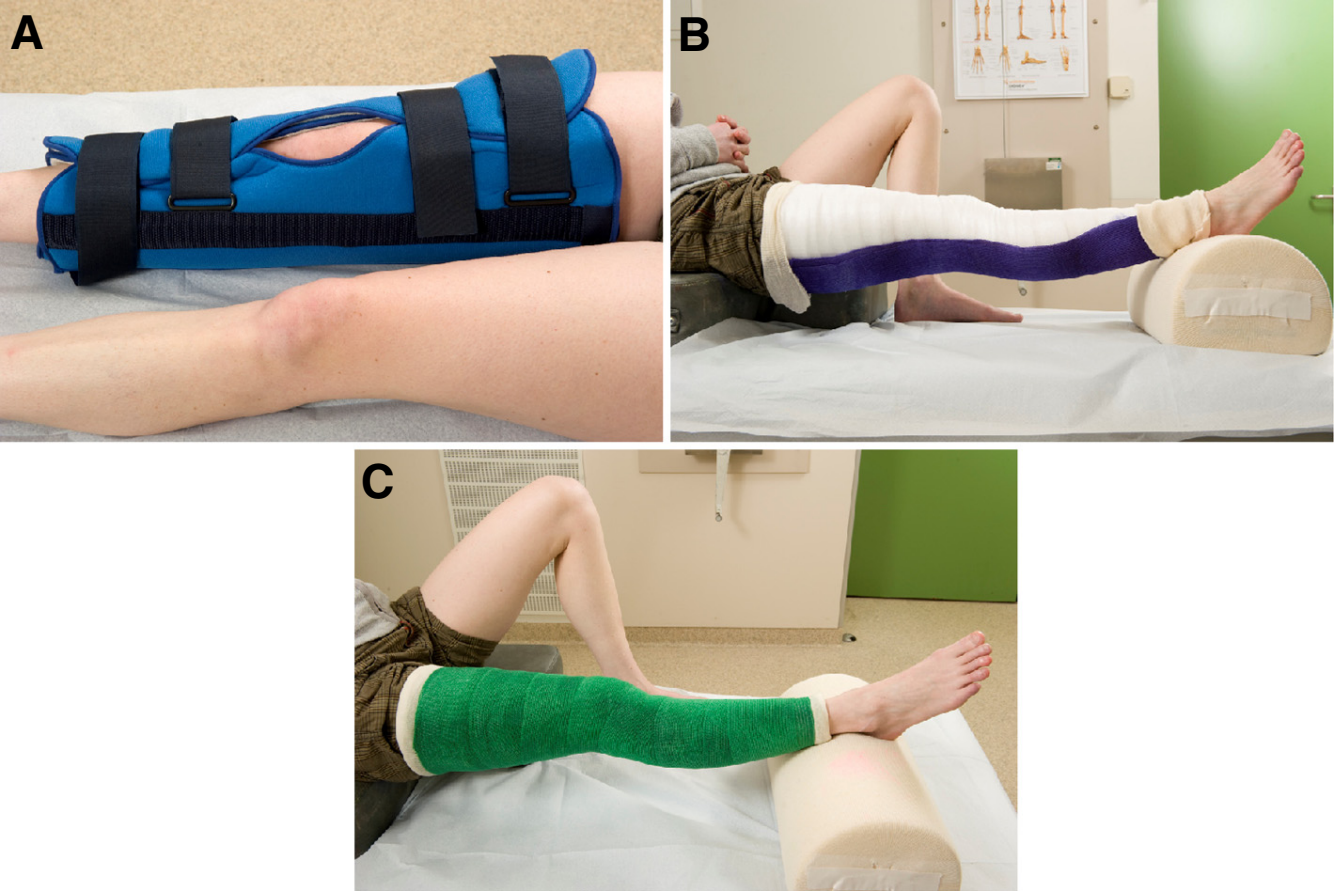
**Conservative treatment in acute primary patella dislocation. A**: brace. **B**: posterior splint. **C**: cylinder cast. Published with permission of the patient.

Patients with patellar dislocations are common in the emergency department (incidence 5.8-7 per 100,000 per year) [1,2]. After closed reduction, acute primary dislocations can be managed conservatively by immobilisation with a cylinder cast, posterior splint or removable knee brace or by surgical treatment (Figure 1).

The most important complications of primary acute patellar dislocations are recurrence and continued disability [3]. Consequently, it is important to determine the best treatment providing patellar stability and knee function. However, no consensus seems to exist on the most appropriate means of conservative treatment. Also, the immobilisation time has not been standardised. We, as emergency physicians, are seeking the best possible way to treat patients with acute primary patellar dislocation in the emergency department (ED). Therefore the primary aim of this review is to answer the following clinical query: Does immobilisation with a cylinder cast provide less redislocation and joint movement restriction than a knee brace or posterior splint in patients with primary acute patellar dislocation? Secondly we assessed the redislocation rates in surgical versus conservative treatment.

## Review

### Methods

**Figure 2:**
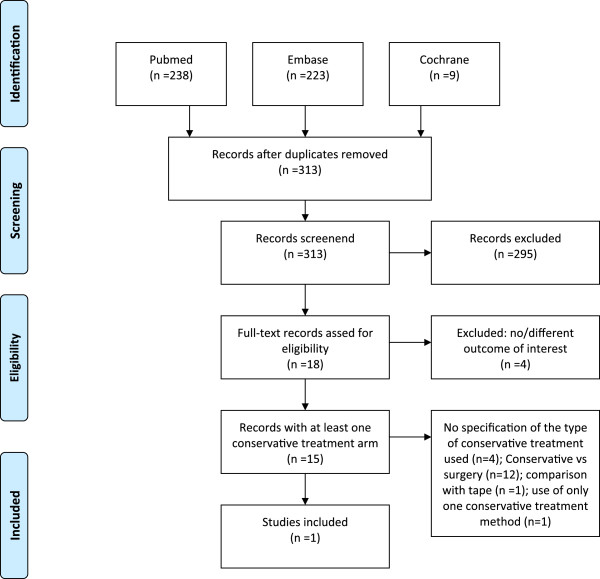
Flow chart review.

A systematic review of the literature on conservative treatment of patellar dislocations was conducted. A literature search of the PubMed, Embase and the Cochrane Library databases was performed on 17 August 2012. The search terms are shown in Table 1. No limits were imposed. Duplicates were removed using Reference Manager. Studies were eligible for inclusion when the target intervention was a cylinder cast compared with a posterior splint and a knee brace in patients with primary acute patellar dislocations. These inclusion criteria were used to make a selection based on the title and/or abstracts. Animal studies, case reports with fewer than five cases and studies on patellar fractures were excluded from the review. Independently and in duplicate, two of the authors performed a more thorough selection using the aforementioned exclusion criteria. Bibliographies of all the selected articles were reviewed for additional articles (Figure 2). The primary objectives were to compare redislocation rates and joint movement restrictions after treatment with cylinder casts, posterior splints and knee braces. Secondary objectives were to compare redislocation rates after surgery and conservative treatment. Results were expressed as relative risks (RR) with 95% confidence intervals (CI95%) or *p* values.

**Table 1:** Search query as used in pubmed, embase and the cochrane library August 17th, 2012

	**AND**	**AND**
patel* OR kneecap	luxat* OR subluxat* OR dislocat* OR displace* OR disarticulat* OR floating	cylinder cast OR gypsum OR plaster OR splint OR immobilisation OR immobilization OR “conservative treatment” OR brace OR sleeve OR support OR bandage OR orthosis OR nonoperative

### Results of the search

The electronic search identified 470 articles (flowchart: Figure 2). After removing the duplicates, 313 articles remained. Eighteen articles were selected by screening the titles and abstracts of the 313 articles. Of these, studies with a different outcome from the one of interest were excluded (*n* = 4). At the end of the selection process, 15 studies with at least one conservative treatment arm remained (Table 2) [3-17]. No additional articles were found after screening the bibliographies of these articles. Of these 15 studies, 4 did not describe the type of conservative treatment used [4,9-11], 12 compared surgical with conservative treatment [3-11,13,15,16], and 1 compared cylinder casts with tape [14]. These studies could only be used as single-arm studies.

**Table 2:** Study results

	**Conservative treatment**		**Comparison**		***p*-value**
**Study, year**	**Method (*n*)**	**Redislocation *n *(%)**	**Method (*n*)**	**Redislocation *n *(%)**	
**Apostolovic, 2011**	Not specified (23)	1 (4)	Surgery (14)	2 (14)	Ns
**Bitar, 2012**	Brace (20)	7 (35)	Surgery (210)	0 (0)	Nm
**Buchner, 2005**	Brace (63)	17 (27)	Surgery (37)	10 (27)	Ns
**Camanho, 2009**	Splint (16)	8 (50)	Surgery (17)	0 (0)	Nm
**Cash, 1988**	Group 1				
	Splint (54)	23 (43)	Surgery (15)	2 (13)	Nm
	Group 2				
	Splint (20)	4 (20)	Surgery (14)	1 (7)	Nm
**Christiansen, 2008**	Brace (35)	7 (20)	Surgery (42)	7 (17)	Ns
**Cofield, 1977**	Not specified (35)	11 (31)	Surgery (13)	0 (0)	Nm
**Hawkins, 1986**	Not specified (20)	3 (15)	Surgery (7)	0 (0)	Nm
**Hing**^**#**^**, 2012**	Not specified (157)	53 (34)	Surgery 182	47 (37)	0.26
**Mäenpää, 1997**	Cylinder cast (60)	23 (38)	Splint (17) Brace (23)	8 (53) 13 (48)	Ns
Palmu*, 2008	Brace (28)	15 (54)	Surgery (36)	18 (50)	Ns
**Rood, 2012**	Cast (9)	0 (0)	Tape (9)	0 (0)	Nm
**Sillanpää, 2008**	Brace (35)	8 (23)	Surgery (26)	5 (19)	0.84
**Sillanpää, 2009**	Brace (21)	6 (29)	Surgery (17)	0 (0)	0.02
**Sillanpää, 2011**	Brace restricted ROM (13)	3 (23)	Brace free ROM (13)	5 (38)	Ns

^#^Cochrane Review.

*Children and adolescents; Nm: not mentioned; Ns: not significant; ROM: range of motion.

Group 1: congenital abnormality extensor mechanism opposite knee.

Group 2: patients with normal opposite knee on examination.

### Primary outcome

Reported redislocation rates ranged between 0-38% in patients treated with cylinder casts, between 4-53% in patients treated with splints and 6-54% in patients treated with braces (Table 2). However, the aforementioned single-arm studies did not provide an answer to our clinical question.

Therefore, only the study of Mäenpää et al. was suitable for answering the clinical question. The results of this study are given in Table 3. The patient numbers were small in the splint (*n* = 17) and brace (*n* = 23) groups compared to the cylinder cast group (*n* = 60). There were no significant differences in baseline characteristics between groups. The follow-up examination was performed at an average of 13 years later (range 6–26 years). The immobilisation time was shorter in the brace group (2 ± 1 weeks) than in the splint group (3 ± 2 weeks) and the cylinder cast group (4 ± 11 weeks). Redislocation rates ranged from 38% in the cylinder cast group to 47% in the posterior splint group to 57% in the brace group. There were no significant differences among these groups. Furthermore, the redislocation frequencies per follow-up year were significantly higher in the brace group (0.29; *P* < 0.05) than in the cylinder cast group (0.12) and the posterior splint group (0.08). There were no significant differences between the loss of extension and flexion between groups. However, in the cylinder cast group the proportion of loss of flexion and extension was the highest [12].

**Table 3:** Study results: Mäenpää et al. 1997

**Treatment arm (*n*)**	**Redislocation**		**Redislocation/years**		**Loss of extension**		**Loss of flexion**	
	**(%)**	**RR (CI 95%)**		***p***	**(%)**	**RR (CI 95%)**	**(%)**	**RR (CI 95%)**
Cylinder cast (60)	38		0.12		15		27	
Posterior splint (17)	47	1.2 (0.68-2.23)	0.08	Nm	6	0.39 (0.05-2.89)	6	0.22 (0.03-1.55)
Brace (23)	57	1.5 (0.91-2.39)	0.29	< 0.05	13	0.87 (0.26-2.93)	17	0.65(0.24-1.74)

*RR*, relative risk; (CI 95%), 95% confidence interval; *Nm*, not mentioned.

### Secondary outcome

Twelve studies reported redislocation rates after surgery compared to conservative treatment [3-11,13,15,16]. Only one study showed a significantly lower redislocation rate in the surgical group [16]. The other studies did not report a significant difference between surgical and non-surgical management.

### Discussion

The query in the Medline, Embase and Cochrane databases resulted in only one relevant article (Mäenpää et al.). Mäenpää et al. recommend using a posterior splint for acute primary patella dislocation because of the low knee joint restriction and low redislocation rates per follow-up year. They did not find a significant difference in the redislocation frequency among the cylinder cast, splint and brace groups. In contrast, they showed that patients treated with a brace exhibited a significantly higher redislocation frequency per follow-up year. This effect might be due to the shorter immobilisation time in the brace group compared to the other groups. Another explanation might be found in the type of brace used: simple straps and knee sleeves. These days knee braces that maintain better patellar alignment are available.

Mäenpää et al. showed the highest frequency of knee joint restriction in patients treated with cylinder casts. However, the difference to the other groups was not significant. This might be the result of the limitation of joint movement caused by the cylinder cast, which might protect against redislocation but may cause degenerative changes in the bone, cartilage and knee ligaments. Moreover, this movement limitation might also be caused by the longer immobilisation time in the cylinder cast group compared to the splint and brace groups. The lack of standardisation of the immobilisation time between groups in the Mäenpää et al. study makes the results unconvincing. Therefore, to find the most appropriate treatment for patellar dislocation, special attention should be given to the immobilisation time.

Although the above-mentioned study represents the best available evidence, the evidence level remains low because of the small study population, difference in immobilisation duration between groups, use of old braces and limitations in the study design.

Furthermore, 11 out of 12 studies comparing surgical and conservative treatment did not report significantly different redislocation rates [3-11,13,15,16]. In conformation with these results, a recently published Cochrane review based on five studies involving 339 participants did not find evidence of lower redislocation rates in patients who were managed with surgical repair compared with those who were managed with conservative treatment [11].

## Conclusion

Based on the best available evidence, the treatment for primary acute patellar dislocation remains controversial. A posterior splint might be the best therapeutic option because of the low redislocation rates and knee joint restrictions. However, this recommendation is based on only one small study with significant limitations. Further investigation with modern braces and standardisation of immobilisation time is needed to find the most appropriate conservative treatment for patellar dislocation.

Furthermore, there is insufficient evidence to confirm the added value of surgical management.

## Competing interests

The authors declare that they have no competing interests.

## Authors’ contributions

All authors contributed to the research and the work presented in this paper. JPG: first author- conceived the research question, carried out the literature search, critical appraisal and data extraction, discussion and manuscript writing. LMV: second authors- carried out the literature search, critical appraisal, data extraction and participated in writing the manuscript. RAPAH: third author- supervised the developing of the research question, the discussion, and the manuscript writing. MIG: fourth author- primary supervisor. Coordinating the research project, supervising the discussion, manuscript writing and editing. All authors read and approved the final manuscript.
